# Cells-Based Drug Delivery for Cancer Applications

**DOI:** 10.1186/s11671-021-03588-x

**Published:** 2021-09-03

**Authors:** Ying Du, Shujun Wang, Meilin Zhang, Baoan Chen, Yanfei Shen

**Affiliations:** 1 Department of Hematology and Oncology (Key Department of Jiangsu Medicine), Zhongda Hospital, School of Medicine, Southeast University, Ding JiaQiao Street 87, Nanjing, 210009 People’s Republic of China; 2 Department of Chemistry and Chemical Engineering, Southeast University School of Medicine, Ding JiaQiao Street 87, Nanjing, 210009 People’s Republic of China

**Keywords:** Cell, Drug delivery system, Tumor

## Abstract

The application of cells as carriers to encapsulate chemotherapy drugs is of great significance in antitumor therapy. The advantages of reducing systemic toxicity, enhancing targeting and enhancing the penetrability of drugs to tumor cells make it have great potential for clinical application in the future. Many studies and advances have been made in the encapsulation of drugs by using erythrocytes, white blood cells, platelets, immune cells and even tumor cells. The results showed that the antitumor effect of cell encapsulation chemotherapy drugs was better than that of single chemotherapy drugs. In recent years, the application of cell-based vectors in cancer has become diversified. Both chemotherapeutic drugs and photosensitizers can be encapsulated, so as to achieve multiple antitumor effects of chemotherapy, photothermal therapy and photodynamic therapy. A variety of ways of coordinated treatment can produce ideal results even in the face of multidrug-resistant and metastatic tumors. However, it is regrettable that this technology is only used in vitro for the time being. Standard answers have not yet been obtained for the preservation of drug-loaded cells and the safe way of infusion into human body. Therefore, the successful application of drug delivery technology in clinical still faces many challenges in the future. In this paper, we discuss the latest development of different cell-derived drug delivery systems and the challenges it will face in the future.

## Introdution

Cancer is still an insurmountable disease in the medical field. Chemotherapy is the main treatment for cancer. However, the systemic toxicity and drug resistance brought by chemotherapy drugs lead to the increase of failure rate [1]. In recent years, researchers have focused on the study of drug encapsulation by cells or nanoparticles (NPs). Intravenous administration of chemotherapeutic drugs will have great side effects on human body. At the same time, many drugs have poor penetration and targeting to tumor cells. Poor water solubility of chemotherapeutic drugs is also a common clinical problem. Fortunately, the emergence of new drug loading technology may solve these problems [2]. Drugs are usually infiltrated into cells by the hypotonic method when using cells to load drugs. However, the change of permeability will lead to deformation of cells and reduce the stability of cell membrane. The combination of NPs and cells can improve drug encapsulation and release, which has become a hot research topic in recent years [3, 4]. Increasing the biocompatibility of NPs, enhancing the ability of tumor targeting and prolonging the circulation are the main advantages of cell-derived vector. Among them, the enhancement of targeted drug delivery has become an important concern [5]. In order to solve this problem, researchers modify the cell surface to improve the targeting of DDS to tumor tissue, improve the drug concentration in tumor tissue and achieve effective tumor inhibition effect finally [6]. This review introduces the development of encapsulation of drugs or NPs with erythrocytes, platelets, immune cells, tumor cells and stem cells (Table 1) [7–35].

**Table 1:** Application of drug delivery technology based on different cells in cancer

Cell	Encapsulated NPs	Drugs	Types of tumors	References
Erythrocyte				
	PLGA NPs	Curcumin	Liver cancer	Xie et al. [7]
	MnFe_2_O_4_ NPs	IR-780		Sousa Junior et al. [8]
	Fusing tumor cell membrane-associated antigens with nanoerythrosomes	aPD-L1	Breast cancer, melanoma	Han et al. [9]
	PB NPs	Gamabufotalin	Breast cancer	Liu et al. [10]
		siRNA, DOX	Breast cancer	Wang et al. [11]
	PB/manganese dioxide NPs	DOX	Breast cancer	Peng et al. [12]
	PLGA NPs	DOX	AML	Aryal et al. [13]
	Mesoporous silica NPs	Ce6, DOX	Breast cancer	Su et al. [14]
	Metal–organic framework NPs	Glucose oxidase Tirapazamine	Colon cancer	Zhang et al. [15]
	PLGA NPs	DOX	Lymphoma	Luk et al. [16]
Platelet				
	Chitosan oligosaccharide–PLGA copolymer	Bufalin	Liver cancer	Wang et al. [17]
	Liposome	DOX	Breast cancer	Liu et al. [18]
	PLGA NPs	ICG, aPDL1	Breast cancer	Han et al. [19]
		DOX	Lymphoma	Xu et al. [20]
		Epirubicin	Myeloma	Dai et al. [21]
Immunological cell				
Macrophage				
		DOX	Lung cancer	Evangelopoulos et al. [22]
	Gold nanorods	Liposome DOX	Breast cancer	Nguyen et al. [23]
	Au nanoshells	DTX	Breast cancer	Choi et al. [24]
	PLGA NPs, Fe_3_O_4_ magnetic NPs		Colon cancer, breast cancer	Han et al. [25]
Cytotoxic T cells				
	PLGA NPs	Taxol	Gastric cancer	Zhang et al. [26]
Leukocyte				
		DOX	Breast cancer melanoma	Molinaro et al. [27]
NK cell				Deng et al. [28]
		TCPP	Breast cancer	
Tumor cell				
	PLGA NPs	ICG	Breast cancer	Chen et al. [29]
	Upconversion NPs		Breast cancer	Rao et al. [30]
	PLGA NPs	Cancer cell membrane fractions	Glioblastoma	Jin et al. [31]
	Magnetic iron oxide NPs	DOX	UM-SCC-7	Zhu et al. [32]
Stem cell				
	AlPcS4@FNPs		Osteosarcoma	Lenna et al. [33]
		Ce6	Lung melanoma	Ouyang et al. [34]
	Polydopamine NPs Gelatin nanogels	DOX	Cervical carcinoma	Gao et al. [35]

### Erythrocyte

**Fig. 1:**
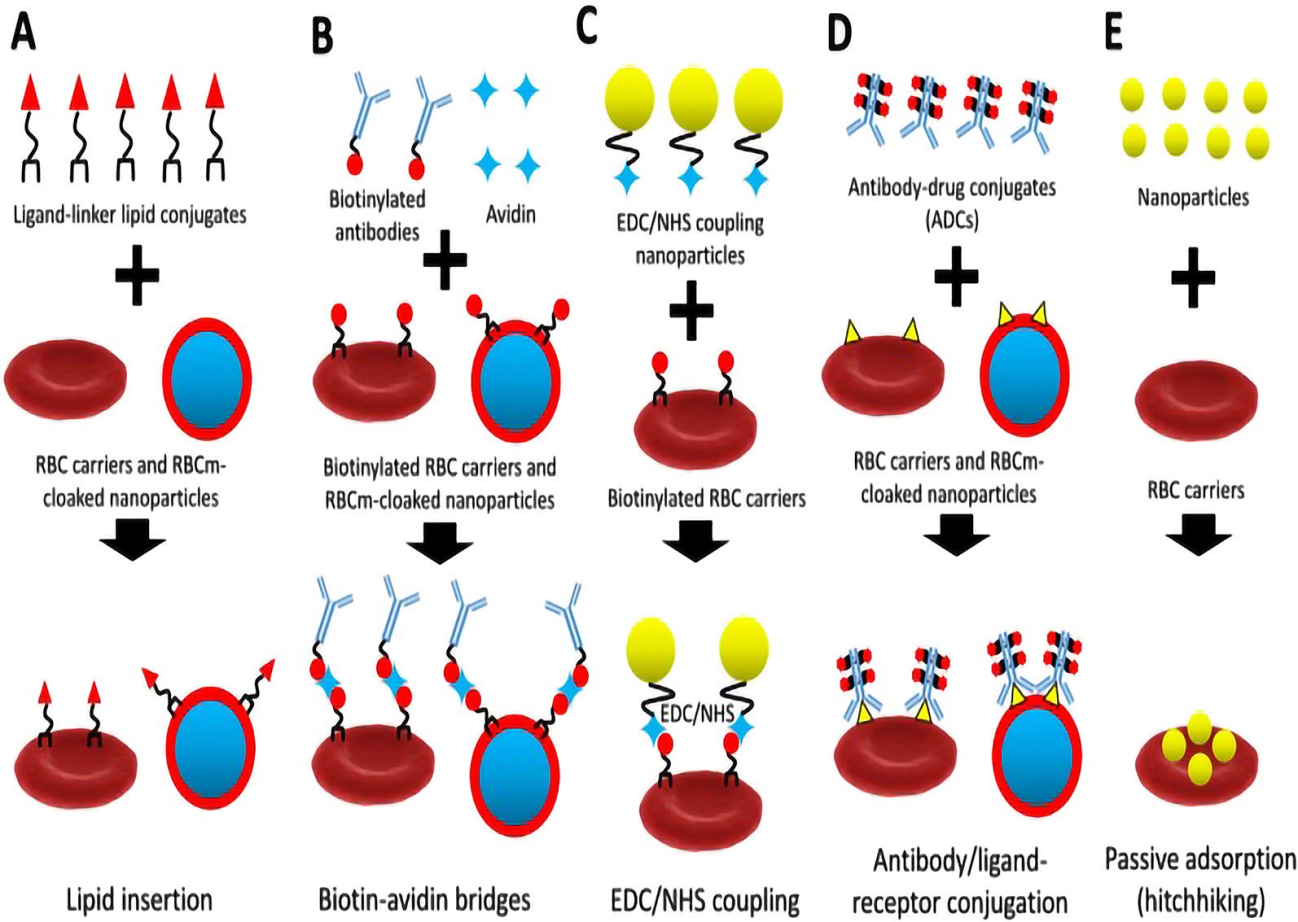
Schematic diagram of binding method between erythrocyte membrane and NPs (**A**), biotin-avidin bridges (**B**), EDC/NHS coupling (**C**), antibody/ligand-receptor conjugation (**D**), and passive adsorption (hitchhiking) (**E**) methods for refunctionalization of erythrocyte-based nanomedicine. Reprinted with permission from Ref. [30]. Copyright © 2019, Theranostics

Erythrocytes are regarded as a promising cell-mediated drug delivery platform due to the inherent advantages of biocompatibility, long life span and easy access [7, 36]. Initially, erythrocytes often encapsulate drugs directly. With the development of research, erythrocytes combined with functional NPs were appeared. The transformation of single drug chemotherapy to targeted therapy, immunotherapy, photothermal therapy (PTT) and so on has been realized. Erythrocyte membrane can combine with NPs in many ways. Sun et al. summarized several technologies of erythrocyte membrane carrier constructed by covalent and noncovalent binding methods including lipid insertion, biotin-avidin bridge, EDC/NHS coupling, antibody/ligand–receptor conjugation and passive adsorption (Fig. 1) [36]. Wang et al. studied the erythrocyte-cancer cell hybrid membrane to achieve drug delivery. The hybrid membrane not only has the ability of immune camouflage, but also has the ability of tumor targeting [4]. The delivery efficiency of therapeutic NPs based on tumor passive targeting strongly depends on the proper regulation of blood circulation time or tumor microenvironment. Based on this theory, Sousa Junior et al. proposed a new scheme of camouflaged magnetofluorescent nanocarrier (MMFn) by erythrocyte membrane. The blood circulation time of MMFn is up to 92 h, and it has high transfer efficiency, which has been verified in the mouse tumor model [8]. The objective clinical development of cancer vaccine is limited. This is related to the high level of programmed death ligand-1 (PD-L1) expression in tumor cells, which leads to the immunosuppressive characteristics of tumor microenvironment. The expression of PD-L1 on antigen-presenting cell (APCs) can induce T regulatory cells [37]. Hence, the combination of anti-PD-L1 (aPD-L1) and cancer vaccine may be beneficial. Aging erythrocyte has a unique ability to target spleen antigen-presenting cells. Therefore, the combination of aPD-L1 blockade with nano-erythrocytes can cause antigen reaction in vivo, which can valid inhibit tumor growth and reduce tumor metastasis [9]. PTT is a new method for the treatment of tumor, which has great development potential and will become an important method for the treatment of tumor. Prussian blue (PB) NPs with high photothermal conversion, strong blue, magnetic, good biocompatibility and stability are considered to be suitable for cancer PTT. However, the blood retention time of PB NPs is short, which makes its antitumor effect reduced greatly. The CD47 on the surface of erythrocytes is the self-recognition protein of reticuloendothelial system in vivo, so the nanomaterials coated with erythrocytes can improve the immune escape ability and prolong the half-life in the circulation. The erythrocyte membrane was used to improve the drug accumulation of PB in the tumor site [10]. As a new drug delivery system (DDS), extracellular vesicle (EV) carrier has the advantages of safety, high efficiency and long circulation. However, the disadvantages of low yield, high cost and heterogeneity limit the application of EV [38]. The preparation of membrane-derived artificial vesicles by fracture and self-assembly has become an effective strategy to overcome the problem. The size and uniformity of the multifunctional mimic vesicles derived from red blood cells can be well controlled. The researchers used erythrocyte-derived mimic vesicles (MVs) loaded with P-glycoprotein (P-gp) siRNA and Doxorubicin (DOX) for the targeted treatment of multidrug-resistant tumors. This MV-based DDS provide a new direction for the collaborative targeted therapy of tumor [11]. The transport platform derived from erythrocytes can be designed by mechanical operation, which can make the diameter from micrometer to nano. The research results indicate that the real-time circulation dynamics of erythrocytes vector is related to its diameter. The researchers designed a micro- and nano-erythrocytes optical carrier based on the real-time fluorescence imaging of particles in the subcutaneous vascular system of healthy mice and studied the circulation dynamics. The result suggests that the average emission half-life of the micro- and nanocarriers in the blood vessels was ~ 49 min and 15 min, respectively. After 7 days, the second injection of particles increased the average emission half-life of the microsize carrier to 1 h, and the variable half-life of nanocarriers ranged from 25 to 60 min [39]. Phosphatidylserine exposed on the carrier surface is an important mechanism for their removal from the circulation [40]. The emission half-life of NPs in blood is shorter after the first injection, which indicates that macrophages may be more effective in removing these particles when mediated by the mechanism of phosphatidylserine surface exposure. The shape and deformation characteristics of the microsize carriers contribute to the cycle time of these particles. These biochemical and biomechanical characteristics are important factors for the safe and effective translation of the red blood cell-derived platform [39]. Therefore, these series of research results show that it is necessary to carry out more detailed research on erythrocytes-derived vectors. Only in this way, can we understand the mechanism of the carrier acting on the disease and use these mechanisms to design better erythrocytes-derived carrier.

### Platelet

**Fig. 2:**
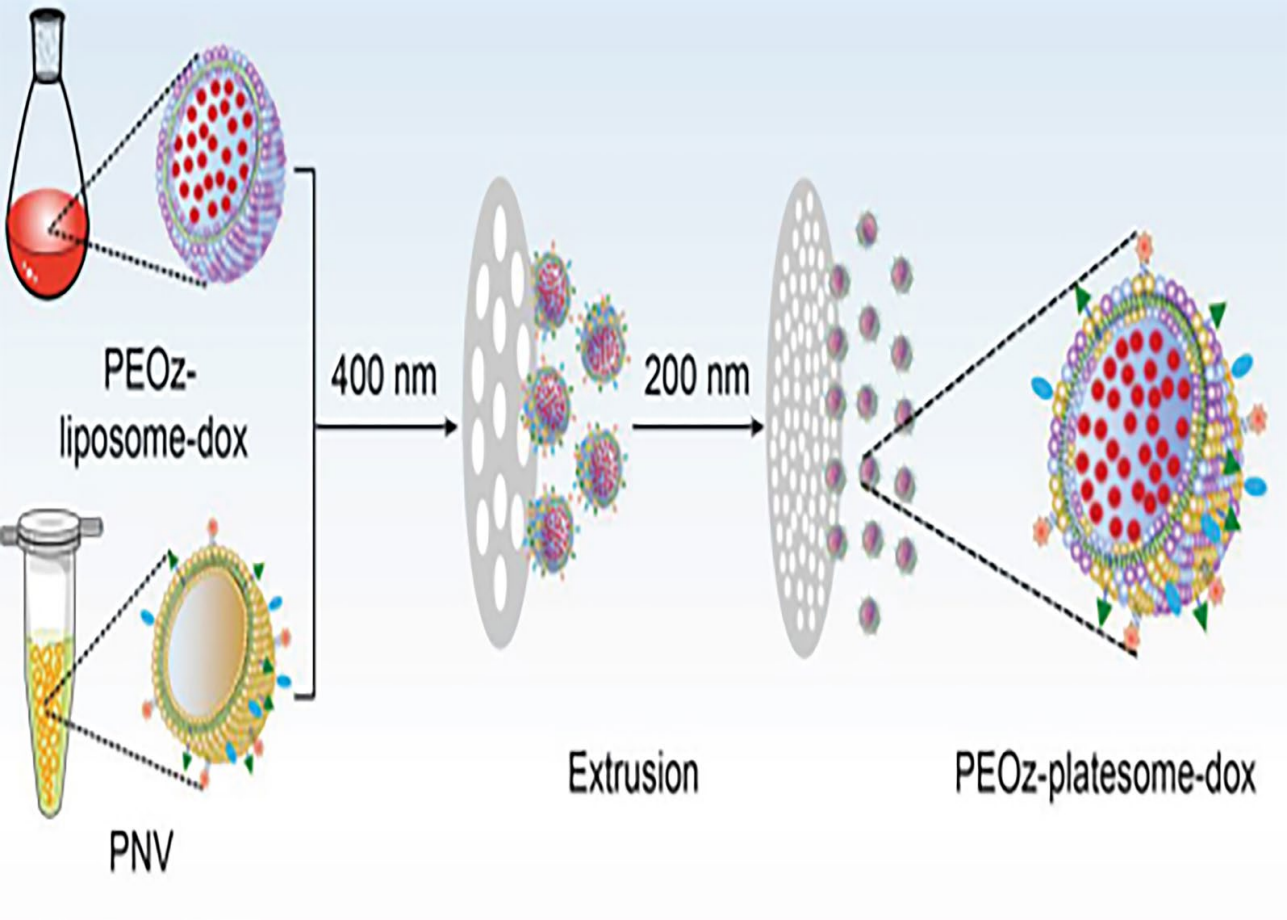
Schematic illustration of the preparation of PEOz-platesome-dox. PEOz-platesome-dox was generated by coextrusion of PEOz-liposome-dox and PNV. Reprinted with permission from Ref. [14]. Copyright © 2019, Wiley

Platelet is a kind of blood cell without nuclear. The life span of platelets is about 8–10 days. Compared with the long circulating time of erythrocytes for four months, the proper circulating time of platelets can avoid unnecessary accumulation in vivo [41]. The CD47 membrane protein of platelets can send a "don't eat me" signal to macrophages to avoid phagocytosis by macrophages. Therefore, platelet biomimetic DDS is expected to improve the escape of macrophages in vivo and enhance the retention in tumor tissue [17]. Studies have indicated that platelets play an important role in promoting tumor cell proliferation, tumor vascular integrity and tumor cell invasion due to their multiple angiogenic regulators. Platelet-derived microparticles (P-MPs), such as vascular endothelial growth factor (VEGF), matrix metalloproteinase (MMP), epidermal growth factor (EGF) and platelet-derived growth factor (PDGF), can induce the activation of MAPK and AKT signaling pathways in tumor cells, so as to stimulate the over expression of proteins required for tumor cell proliferation [42]. Platelet aggregation is related to circulating tumor cells (CTC). In addition, Platelets can prevent vascular extravasation of CTC by enhancing the adhesion between platelets and endothelial cells. More importantly, platelets can protect CTC from immune surveillance of natural killer cells. Hence, it is promising to establish platelet membrane-based DDS using the advantage of platelet targeting to tumor cells. The common method of preparing the platelet membrane is ultrasound and repeated freeze–thaw [41]. In an earlier study on platelet-encapsulated NPs, Hu et al. showed that platelets can encapsulate loaded NPs effectively. Platelet-derived vector has strong targeting capability to injured blood vessels and tumors and plays a good therapeutic role in disease [43]. Liu et al. developed a pH responsive platelet membrane-lipid hybrid drug carrier for tumor targeted delivery. The drug loading platform system showed longer plasma half-life and enhanced tumor accumulation when doxorubicin is triggered and released into mouse tumor models. This result is superior to the traditional pH sensitive liposome (Fig. 2) [18]. The platelet DDS combines immunotherapeutic drugs with photothermal agents. In the latest research, platelet DDS combines immunotherapeutic drugs with photothermal drugs. Drug-modified platelets are recruited to the tumor site to further activate the anticancer immune response, inhibit the growth of residual tumor and improve the survival rate. This is due to inflammation and injury at the tumor site after thermal ablation [19]. The existing researches on platelet-derived nanocarriers show that platelet-mediated DDS has broad prospects.

### Immunological Cell

**Fig. 3:**
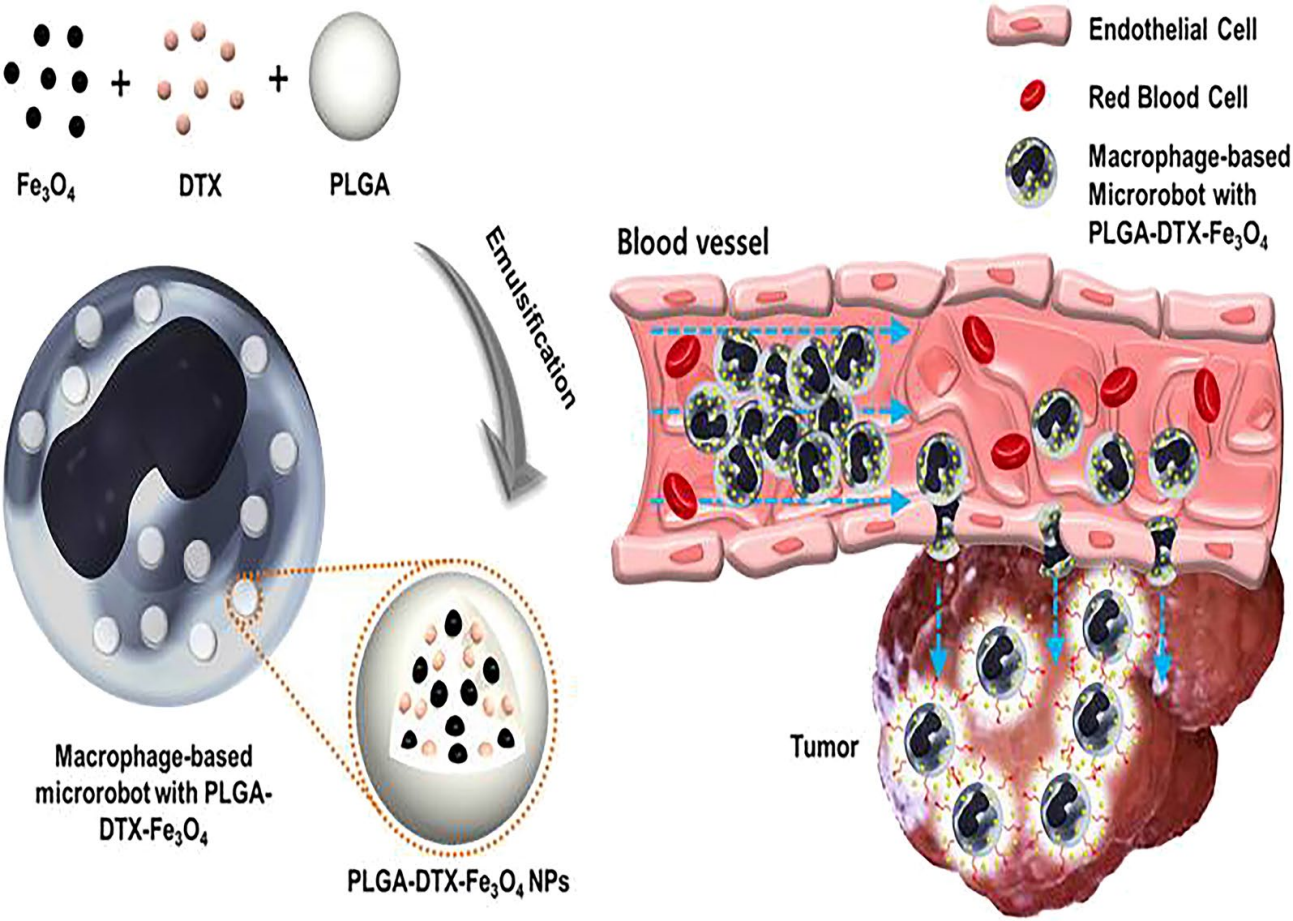
Schematic diagram of the macrophage-based microrobot with PLGA-DTX-Fe_3_O_4_ (left). Describe the targeting and treatment of tumors in the in vivo environment (right). Reprinted with permission from Ref. [20]. Copyright © 2016, Sci Rep

At present, many people focus on the surface modification of NPs carriers to match the biological environment and complexity. However, mononuclear phage system has the ability to recognize and isolate foreign substances, which leads to the decrease in drug concentration in tumor site [25]. NPs can also deliver drugs to tumor areas according to enhanced permeability and retention, but the permeability of tumor vessels is often affected by vascular structural abnormalities and blood supply heterogeneity. Another reason we have to consider is that the size and charge of NPs also affect the amount of them entering the tumor. For example, NPs larger than 100 nm in diameter can be phagocytized by macrophages or filtered by the liver [22]. Therefore, the existence of these factors often leads to NPs cannot enter the tumor site efficiently. Immune cells play an important role in tumor targeting and preventing drug diffusion to normal tissues. Macrophages become one of the ideal drug carriers for DDS because of their natural phagocytic ability, blood barrier and chemotaxis to solid tumors. NPs encapsulated by macrophages will enhance the permeability of NPs to tumor tissue. And the antitumor effect will be further enhanced when the antitumor drugs are combined with photothermal therapy, which has been confirmed in a certain research (Fig. 3) [23]. Monocytes are considered to be the precursor of macrophages, with the ability of phagocytosis, elimination of injured cells and participation in immune response. Moreover, monocytes are the largest cells in the blood which make it easy to load therapeutic NPs. They will differentiate into macrophages when monocytes are recruited to the tumor site. NPs encapsulated by macrophages will then migrate and chemotactic to the anoxic area of the tumor [24, 44].

Cytotoxic T lymphocytes (CTL), a subdivision of white blood cells, are specific T cells that secrete various cytokines to participate in immune function. It has killing effect on virus and tumor cells and forms an important defense line against virus and tumor together with natural killer cells. CTL, also known as killer T lymphocyte, is an important part of the antitumor of body and one of the main effect cells of tumor immunotherapy. It has long blood circulation time and high expression of adhesion molecules. Therefore, CTL has the ability to recruit and locate the tumor sites. All of these advantages lay the foundation for treatment tumor with CTL-derived NPs [26].

Leukocytes are a kind of colorless, spherical and nucleated blood cells. The total number of leukocytes in normal adults was (4.0–10.0) × 10^9^/L [45]. When inflammation occurs, leukocytes were activated to fight bacteria or viruses, which is a manifestation of their important role in the immune system. Leukocytes participate in various immune reactions, cell interactions and have the ability to migrate [46]. They are ideal carriers of chemotherapeutic drugs and regulatory factors of tumor microenvironment (TEM), which are mainly due to the homing characteristics of leukocytes in inflammatory and tumor sites. Roberto Molinaro et al. used the targeting characteristics of leukocyte to tumor-related blood vessels with inflammation to deliver DOX to mouse models with breast cancer and melanoma effectively. The results showed that DOX-loaded leukocytes showed stronger antitumor activity in reducing tumor volume and prolonging survival time [27, 47]. Neutrophils are the most abundant type of leukocytes, accounting for 40–75% of the total number of leukocytes. What's more, neutrophils are the first cell type to reach the site of inflammation. The accumulation of neutrophils mediated DDS in tumor site is further enhanced when photothermal therapy triggers tumor site inflammation [48]. In noncancer diseases, the migration characteristics of neutrophils also provide a new idea for disease treatment. Research show that neutrophils can selectively carry cRGD liposomes as cell carriers, when cerebral ischemia occurs. Thus, neutrophils enter the blood–brain barrier and penetrate into the brain parenchyma, and eventually deliver the drug to the site that needs treatment [49]. CTCs in the blood are easy to proliferate and form metastasis in anatomically distant organs. However, it is difficult to be targeted therapy because of the circulatory state and low concentration of CTCs. Fortunately, they are easy to gather near endothelial cells which is similar to the migration characteristics of white blood cells. The main reason is that CTC is similar to leukocyte in volume and shape, so it is also surrounded by endothelial cell wall when blood flows. This kind of edge phenomenon can surround CTC in white blood cells effectively. In other words, leukocytes are also potential therapeutic vectors for CTC [50].

Natural killer cell (NK) is an important immune cell in the body, which is not only related to antitumor, antivirus infection and immune regulation, but also involved in hypersensitivity reaction and autoimmune diseases in some cases. NK can recognize target cells and killing media. Unlike T and B cells, it is a kind of lymphocyte that can kill tumor cells and virus-infected cells without pre sensitization [51]. Deng et al. found that NK-derived NPs have targeted effect on tumor according the proteomic analysis of NK cell membrane. It is more satisfactory that NK cells can also induce or enhance the polarization of pro-inflammatory M1 macrophages, thus producing antitumor immunity. Supported by this theory, Deng and other researchers encapsulated photodynamic agents in NK cells. The result showed that NK cell DDS showed a strong immunotherapeutic effect in the antitumor process. It not only can inhibit the growth of primary tumor effectively, but also has obvious inhibitory effect on distant tumor [28].

### Cancer Cell

**Fig. 4:**
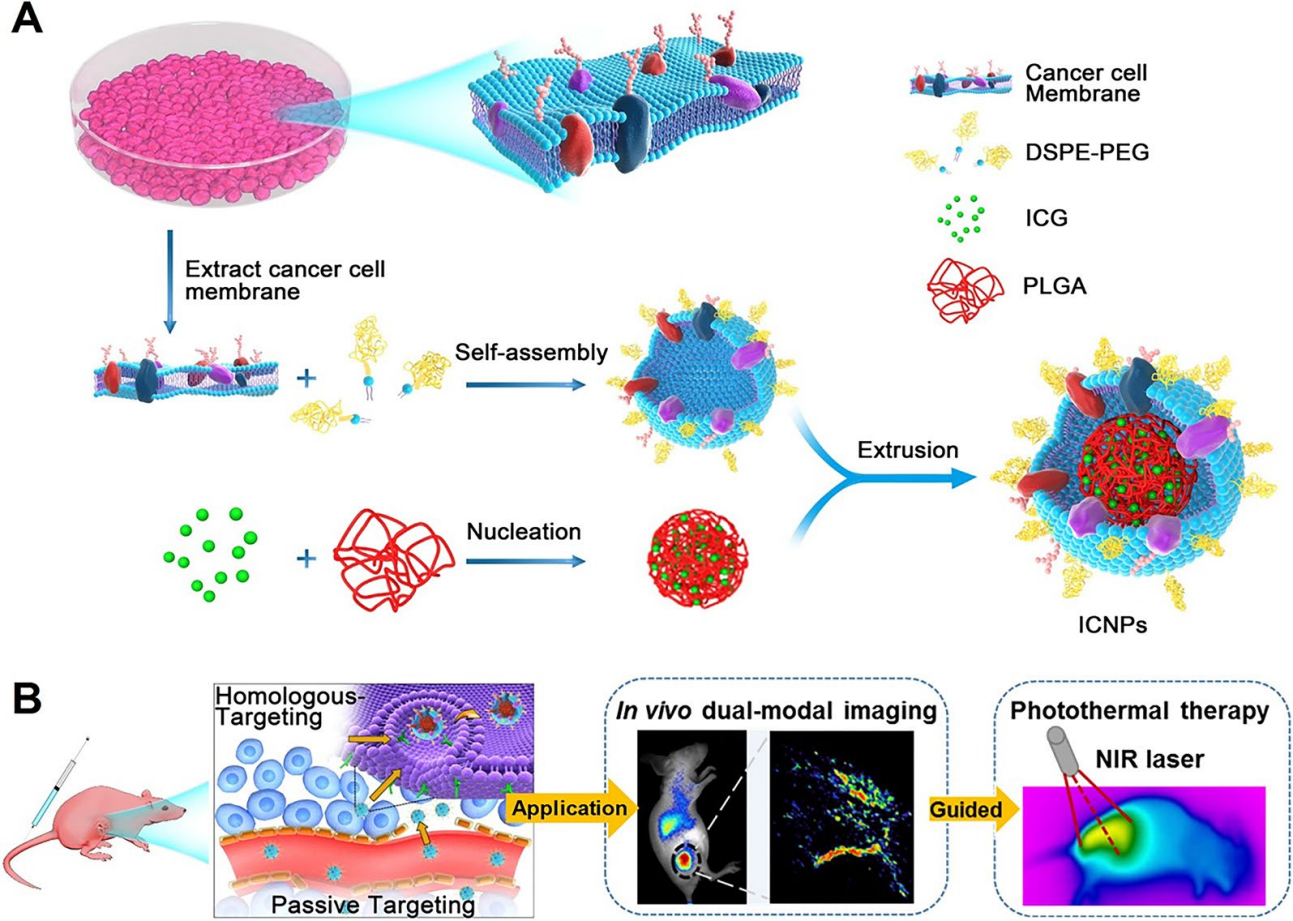
Illustration of the cancer cell membrane–biomimetic NPs for targeting recognition of source cancer cell, dual-modal imaging, and photothermal therapy. (**A**) Preparation procedure of ICNPs. Extracting cancer MCF-7 cell membrane hybridized with PEGylated phospholipids (DSPE-PEG) and then coated onto ICG-loaded polymeric cores by extrusion. (**B**) Schematic of homologous targeting ICNPs for dual-modal imaging guided photothermal therapy. Through specific homologous targeting and the EPR effect (passive targeting), ICNPs realized perfect tumor accumulation, dual-modal FL/PA imaging, and effective photothermal therapy after intravenous injection. Reprinted with permission from Ref. [24]. Copyright © 2016, ACS Publication

The infinite ability of cancer cells to replicate is a nuisance, but the ability of cancer cells to resist cell death allows them to overcome immune clearance [30]. Cancer cells that express surface adhesion molecules such as epithelial cell adhesion molecules, n-cadherin and galectin-3 have homologous adhesion domains, which can promote the occurrence of multicellular aggregation. The ability to homologous bind to membrane proteins leads to the use of cancer cells for surface functionalization of NPs. Chen et al. used cancer cell membrane to encapsulate ICG (Indocyanine green)/poly (lactic co glycolic acid) (PLGA) core and cancer cell membrane shell to achieve synchronous targeting and tumor treatment. The results showed that this method not only had high homologous targeting effect at the cell level, but also had superior spatial resolution and deep penetration at the animal level (Fig. 4) [29]. Studies have shown that mitochondria play an important role in regulating the proliferation of cancer cells. The initiation of apoptosis in mitochondria leads to the release of cytokines such as cytochrome c, which leads to a cascade of cytosolic activity and cell death [52]. Mitochondrion is also closely related to multidrug resistance (MDR) of cancer [53]. Targeted intervention of mitochondrion will become a promising strategy for cancer treatment. Accumulation of mitochondrial targeted drugs can trigger mitochondrial apoptosis pathway, leading to programmed cell death and cancer cell suicide. Studies have shown that metal oxides such as zinc, copper and ceria can inhibit the proliferation of cancer cells in a variety of cancer cell lines through this mechanism. Therefore, some researchers have developed nanoparticles targeting mitochondria. For example, Zn-doped CuO NPs (TPP-ZC-IR-PNPs) and co-loaded with docetaxel and lonidamine (cl-M/DL) system studied by Ruttala et al. All of them have strong targeting ability and therapeutic effect in tumor therapy [52, 54].

### Stem Cell

Tumor can emit chemokines to recruit mesenchymal stem cells (MSCs) to form supporting matrix for tumor growth [55]. MSC is a kind of pluripotent stem cell, which has the ability of self-renewal, multi-directional differentiation and regulation of immune response. It was isolated from brain, lung, liver, kidney, cord blood, placenta and other tissues successfully. It is speculated that MSCs may be able to migrate to the site of injury or inflammation and repair the injury by producing tissue-specific cells and/or releasing paracrine factors. MSCs can also secrete immunomodulatory soluble molecules to regulate immunity and is mainly used in the treatment of Crohn's disease, liver failure, fibrosis and other diseases. The presence of MSCs in the microenvironment of malignant tumors is helpful for tumor growth and metastasis [56]. However, some studies have shown that MSCs derived from umbilical cord can inhibit the growth of pancreatic cancer cells in mice [57, 58]. MSCs can efficiently transduce adenovirus, lentivirus, mouse retrovirus and other major viral vectors. The transformed and gene-modified MSCs can be widely expanded in vitro which makes them become the ideal vector of gene transfer [59]. Tumor formation requires new cells to support it, just like developing or damaged tissue. The migration of MSCs to tumor tissue makes it widely used as a carrier of antitumor drugs. Stefania Lenna et al. have made achievements in the inhibition of osteosarcoma growth based on the photodynamic technology of MSCs loaded with NPs. They prepared Chlorin e6 (Ce6)-coupled polydopamine NPs (PDA-Ce6) were prepared and loaded into MSCs. The MSCs loaded with PDA-Ce6 (MSC-PDA-Ce6) can target and penetrate the tumor. It is worth mentioning that the effective release rate is up to 60% within 72 h. MSC-PDA-Ce6 is a "Trojan horse" like transport through the endocytosis–exocytosis–endocytosis process between MSCs and cancer cells. What's more gratifying is that there may be a certain degree of hope even in the face of small inoperable tumors or drug-resistant patients [33]. Similarly, it has a good effect in the treatment of melanoma [34].

### Development

Proteins on the membrane surface retain the biological function of cell in cell mediated drug delivery technology. As mentioned above, many studies have shown that cell-based drug delivery enhance biocompatibility, targeting capability and immune escape ability, reduce toxic side effects and prevent the formation of protein corona. All of these advantages avoid and reduce the complexity in the preparation of NPs [55]. Nanotechnology has been widely studied in the field of medicine because of its advantages of easy surface functionalization, controllable design and reducing drug side effects. The diameter of NPs used in the studies is similar to that of bacteria, viruses and other pathogens, generally about 100 nm, so the clearance mechanism of reticuloendothelial system in vivo is easy to be activated. Cell-derived NPs reduce the stimulation of macrophages to release cytokines greatly [60]. A serious problem of NPs is that they will come into contact with plasma proteins when they enter the blood system. Plasma proteins adsorbed on the surface of NPs form protein corona which affects the interaction between NPs and blood components, further leads to increased cell activation and may lead to coagulation or even thrombosis eventually [61]. Therefore, the combination of NPs and cell provides a new prospect for the clinical application of DDS. The progress of DDS is also reflected in the combination of treatment and diagnosis. The addition of ICG and other fluorescent dyes with photothermal effect makes it easy to track drug traces in the process of antitumor cells. At the same time, it is also convenient for tumor imaging and easy to evaluate the therapeutic effect [62]. Unfortunately, the current cell drug delivery technology is only used for preclinical in vitro research. It is not foreseen whether the changes of cell membrane composition and surface modification will lead to immune response when the drug delivery technology used in human body. It is not clear whether the drug can be released into the body in time to produce cytotoxicity, and whether the degradation of NPs in the body will affect human health. These problems will lead to the further study of DDSs based on cells [63].

## Conclusions

Compared with chemotherapy alone, DDS based on cells has the advantages of improving biocompatibility, enhancing immune escape, long circulation and enhancing targeting. At the same time, encapsulation of photosensitizers, chemotherapy drugs and other reagents in the carrier can promote the cooperative treatment of tumor. Erythrocytes, platelets, leukocytes and other cells in DDS have been widely studied and achieved many results. At present, the research on the combination of blood cells and NPs is the most. NPs have the advantages of enhanced permeability and retention effect (EPR), high drug loading efficiency and certain targeting ability, which have clear benefits for tumor therapy.

## Data Availability

Not applicable.

## Abbreviations

NPs: Nanoparticles
PTT: Photothermal therapy
MMFn: Magnetofluorescent nanocarrier
PD-L1: Programmed death ligand-1
APCs: Antigen-presenting cell
PB: Prussian blue
DDS: Drug delivery system
EV: Extracellular vesicle
MVs: Mimic vesicles
P-gp: P-glycoprotein
DOX: Doxorubicin
P-MPs: Platelet-derived microparticles
VEGF: Vascular endothelial growth factor
MMP: Matrix metalloproteinase
EGF: Epidermal growth factor
PDGF: Platelet-derived growth factor
CTCs: Circulating tumor cells
CTLs: Cytotoxic T lymphocytes
TEM: Tumor microenvironment
NK: Natural killer cell
MDR: Multidrug resistance
TPP-ZC-IR-PNPs: Zn-doped CuO NPs
cl-M/DL: Co-loaded with docetaxel and lonidamine
MSCs: Mesenchymal stem cells
Ce6: Chlorin e6
PDA: Polydopamine
ICG: Indocyanine green
EPR: Enhanced permeability and retention effect
